# Notes

**Published:** 1902-11

**Authors:** 


### NOTES.

A Cincinnati, Ohio, paper, in large, black-faced head-lines, records a case of rabies at Washington, Indiana, that occurred twenty-seven years after receiving a dog bite. Such records ought to be most carefully preserved.

The cancer laboratory of the University of Buffalo is very desirous of obtaining from all possible sources live animals, especially the smaller and more manageable ones, with malignant tumors. Rats, dogs, rabbits and fowls are most desired. Such animals may be sent to Dr. H. R. Gaylord, 113 High Street, Buffalo, N. Y. The laboratory will cheerfully pay the expense of transportation. The animals should reach the laboratory alive.

What a single issue of one of the leading magazines of to-day stands for is better conceived by the following facts : The Christmas number of *The Delineator* contains over 230 pages, with 34 full-page illustrations, of which 20 are in two or more colors. The magnitude of this December number, for which 728 tons of paper and 6 tons of ink have been used, may be understood from the fact that 91 presses running fourteen hours a day have been required to print it ; the binding alone of the edition of 915,000 copies represents over 20,000,000 sections which had to be gathered individually by human hands.

Christian Science has extended its field to comparative medicine. One case of a Pekin duck that was unable to step received two truth treatments and fully recovered. Wounds, paralysis, and lung fever have all responded to the treatment.

Dr. Duncan McEachran, of Montreal, Canada, on resigning from the post of Veterinary Medical Inspector for Canada, a position he had held acceptably for thirty-six years, was made " Honorary Veterinary Adviser" as an expression of appreciation of the valuable work he had done for the cattle interests of Canada during his administration. Few men could have served public interests so well for so long a period of time and maintained such cordial interests with the cattle-growers and the government as Dr. McEachran.

The publishers of *Success*, in New York, warn all physicians, dentists, and veterinarians against a swindler who is soliciting subscriptions over the country for their paper without authorlty.

Professor James Law announces the completion of his fourth volume on veterinary medicine. This volume completes the series, which has been in preparation for the past two years.

